# Enhancing crayfish sex identification with Kolmogorov-Arnold networks and stacked autoencoders

**DOI:** 10.1038/s41598-025-34095-z

**Published:** 2025-12-30

**Authors:** Yasin Atilkan, Berk Kirik, Eren Tuna Acikbas, Fatih Ekinci, Koray Acici, Tunc Asuroglu, Recep Benzer, Mehmet Serdar Guzel, Semra Benzer

**Affiliations:** 1 Department of Artificial Intelligence and Data Engineering, Ankara University, Ankara, 06830 Turkey; 2 Department of Biomedical Engineering, Ankara University, Ankara, 06830 Turkey; 3 Department of Petroleum and Natural Gas Engineering, Middle East Techical University, Ankara, 06800 Turkey; 4 Institute of Artificial Intelligence, Ankara University, Ankara, 06560 Turkey; 5 Faculty of Medicine and Health Technology, Tampere University, Tampere, 33720 Finland; 6 VTT Technical Research Centre of Finland, Tampere, 33101 Finland; 7 Department of Management Information System, Ankara Medipol University, Ankara, 06050 Turkey; 8 Department of Computer Engineering, Ankara University, Ankara, 06830 Turkey; 9 Department of Science Education, Gazi University, Ankara, 06500 Turkey

**Keywords:** Crayfish, Sex identification, Deep learning, Machine learning, Kolmogorov-Arnold networks, Stacked autoencoders, Freshwater ecology, Computer science

## Abstract

Crayfish play an important role in freshwater ecosystems, and sex classification is crucial for analyzing their demographic structures. This study performed binary classification using traditional machine learning and deep learning models on tabular and image datasets with an imbalanced class distribution. For tabular classification, features related to crayfish weight and size were used. Missing values were handled using different methods to create various datasets. Kolmogorov-Arnold networks demonstrated the best performance across all metrics, achieving accuracy rates between 95 and 100%. Image data were generated by combining at least five images of each crayfish. Autoencoders were employed to extract meaningful features. In experiments conducted on these extracted features, support vector machines achieved 84% accuracy, and multilayer perceptrons achieved 82% accuracy, outperforming other models. To enhance performance, a novel architecture based on stacked autoencoders was proposed. While some models experienced performance declines, Kolmogorov-Arnold networks showed an average improvement of 3.5% across all metrics, maintaining the highest accuracy. To statistically evaluate performance differences, McNemar’s and Wilcoxon tests were applied. The results confirmed significant differences between Kolmogorov-Arnold networks, support vector machines, multilayer perceptrons, and naive Bayes. In conclusion, this study highlights the effectiveness of deep learning and machine learning models in crayfish sex classification and provides a significant example of hybrid artificial intelligence models incorporating autoencoders.

**Supplementary Information:**

The online version contains supplementary material available at 10.1038/s41598-025-34095-z.

## Introduction

 Crayfish are organisms that play a significant role in freshwater ecosystems and are used as biological indicators^1^. Various species of crayfish generally belong to the Malacostraca class, which includes terrestrial organisms that have adapted to live underwater^2^. These organisms are crucial in assessing water quality and the health of the ecosystem because their bodies are sensitive to environmental changes, providing essential information about water quality^3^.

The narrow-clawed crayfish (*Astacus leptodactylus* Eschscholtz, 1823), also described as synonymous with *Pontastacus leptodactylus* Eschscholtz, 1823^4^, is Turkey’s only significant freshwater crayfish species and is considered one of the most valuable and economically important freshwater crayfish in Europe^4,5^.

The cleanliness of the environment in which crayfish live is directly related to the health and presence of these species. In clean water ecosystems, the presence and health of crayfish indicate that the chemical and physical properties of the water are in good condition^6^. Due to their sensitivity to water pollution, a decline in water quality or ecosystem degradation leads to noticeable changes in the number and health of these organisms. Therefore, monitoring crayfish and assessing their health is an important indicator of ecosystem cleanliness and sustainability^7^. The population dynamics and health status of crayfish provide valuable data for shaping ecosystem management and conservation strategies^8^.

Sex determination in crayfish is important for three main reasons. The first is to understand the reproductive cycles and demographic structures of crayfish populations^9^; the second is to determine fishing strategies and management of crayfish species^10^; and finally, for the systematic classification and taxonomic identification of crayfish species^11^. Sex determination is critical for understanding the reproductive cycles and demographic structures of crayfish populations. During breeding seasons, males and females may exhibit different behaviors, reproductive strategies, and habitat use; therefore, accurate sex determination is essential for reproductive management and understanding population dynamics^9^. Sex determination in crayfish is also important for optimizing fishing strategies and management practices for commercially valuable crayfish species. Protecting female crayfish during breeding seasons is particularly crucial for sustainable fishing practices^10^. Additionally, sex determination is important in the systematic classification and taxonomic studies of crayfish species. Specifically, identifying new species and determining sexual characteristics contribute to understanding biological diversity^11^.

Deep learning and machine learning algorithms have become powerful tools for solving complex problems, revolutionizing many scientific fields in recent years. These technologies, especially when working with large datasets, offer the ability to perform more accurate, faster, and more efficient analyses by surpassing the limitations of traditional methods^12^. Machine learning, and particularly deep learning algorithms, have been successfully applied in various domains such as image recognition, natural language processing, and genetic analysis, paving the way for discoveries and innovations in these fields^13,14^. One of the biggest advantages of these methods is their ability to extract meaningful patterns and features from large datasets without the need for human intervention^12^. As a result, it has become possible to analyze complex data in disciplines such as biology, medicine, and engineering, leading to more accurate predictions. In biological research, in particular, deep learning and machine learning techniques have led to groundbreaking advancements in areas such as species identification^15^, sex determination^16^, disease diagnosis^17^, and the identification of genetic variations^18^. These techniques eliminate the challenges and limitations of traditional methods, enabling the analysis of more complex and large datasets. For example, deep learning applications in image recognition are used to distinguish various species and subspecies, contributing to a better understanding of biodiversity.

Deep learning and machine learning also hold great potential in fisheries research, such as in the sex determination of crayfish^19,20^. These algorithms accelerate the process of automatically identifying and analyzing complex sex characteristics, offering significant advantages in both scientific studies and commercial applications. These technologies are considered revolutionary tools for obtaining critical information necessary for the conservation of biodiversity, management of aquatic ecosystems, and sustainable fishing practices^20^.

Studies on sex determination and species identification in crayfish and other aquatic products highlight the importance of deep learning and machine learning algorithms. For instance, Atilkan et al. (2024) compared deep learning and canonical machine learning models using weight, size, and sex data of healthy and diseased crayfish, along with images, achieving the highest accuracy by combining ResNet50 and RF algorithms^17^. Hasan and Siregar (2021) successfully identified the species, sex, and age of marine crayfish in Indonesia using computer vision techniques^21^. Ye et al. (2023) developed an automated sorting system that classified crayfish size and maturity with 98.8% accuracy using an improved YOLOv5 algorithm^22^. Garabaghi et al. (2022) used a support vector machine (SVM) algorithm to classify healthy and unhealthy freshwater crayfish, evaluating the performance of the SVM model with various kernel functions^19^. Wang et al. (2022) developed a convolutional neural network (CNN)-based system for assessing the freshness of crayfish^23^, while Favaro et al. (2021) explored the potential of support vector machines for detecting the presence of white-clawed crayfish^24^. Chen et al. (2024) improved the SSD model with MobileNetv3 and used the Soft-NMS technique to develop a method for detecting crayfish heads, tails, and claws in real time with high accuracy and speed^25^. Li et al. (2022) applied deep learning in aquatic products for image detection, video detection, species classification, biomass estimation, behavior analysis, and food safety^20^. Zhang et al. (2020) achieved 97.9% accuracy in detecting sea cucumbers (120 samples) using deep learning (Stochastic Gradient Descent (SGD))^26^. Borowicz et al. (2019) developed a system for recognizing whale species in aerial images using deep-learning models^27^. Eickholt et al. (2020) trained deep learning models to automatically identify fish species, thus enabling more effective monitoring and management of fish populations^28^. These studies demonstrate the high accuracy and efficiency of deep learning techniques in sex determination and species classification of crayfish.

These studies and findings emphasize that sex determination in crayfish is not only biologically and ecologically important but also critical from an economic and management perspective. Accurate sex determination plays a fundamental role in understanding the reproductive cycles and demographic structures of crayfish populations, contributing to the optimization of reproductive management and population dynamics. Additionally, the protection of female crayfish during their breeding seasons is necessary to improve the fishing strategies and resource management of commercial crayfish species. In this context, this study aims to achieve sex determination in crayfish using deep learning methods. It is anticipated that deep learning technologies will provide significant advantages in both scientific and commercial applications by making this determination faster, more accurate, and more efficient.

Although machine learning algorithms perform well in classification tasks, several studies have aimed to enhance their performance by modifying key components, combining different classifiers, or employing alternative architectures such as Transformers instead of conventional deep learning models. For example, Kim et al. (2024) proposed a method called Heterogeneous Random Forest, which enhances the diversity — a key strength of the algorithm — to further improve its performance^29^. Nanni et al. (2023) conducted a promising study in the field of medical classification by combining convolutional neural networks with support vector machines through ensemble techniques to achieve improved performance^30^. Xie et al. (2025) proposed a two-stage framework called GAdaBoost, based on the AdaBoost algorithm, to address the label noise problem in classification tasks. The proposed method demonstrated strong performance in terms of robustness and efficiency^31^. Lu et al. (2025) proposed LRAD-ViT, a Vision Transformer–based model for Alzheimer’s disease detection, showing strong diagnostic performance and high computational efficiency^32^. Lu et al. (2025) proposed LAFAN-Net, a deep learning framework for tuberculosis and pneumonia diagnosis that integrates visual and textual information. The model effectively extracts clinically meaningful features, demonstrating its potential for improving diagnostic accuracy in chest X-ray analysis^33^. Lu et al. (2025) proposed CTBViT, a Vision Transformer–based model for tuberculosis classification that focuses on the most relevant image regions while effectively mitigating the overfitting problem^34^.

In this study, we aimed to compare both traditional and recently introduced classification methods for the crayfish sex identification problem using tabular and image-based datasets.

For the binary classification task, conventional machine learning algorithms, including Naïve Bayes, Support Vector Machines, Random Forest, K-Nearest Neighbors, and Artificial Neural Networks, were employed. In addition, a recently proposed method, the Kolmogorov–Arnold Network (KAN), was incorporated to provide a comparative evaluation against these traditional approaches. Furthermore, in the image-based part of the study, autoencoder and stacked autoencoder architectures based on convolutional neural networks were utilized as feature extraction mechanisms, and their performances were systematically compared across the same classification models.

To the best of our knowledge, our study is the first to use Kolmogorov-Arnold networks and autoencoders for sex classification in crayfish. Additionally, a unique feature extraction mechanism was developed by utilizing multiple autoencoders, and this architecture has significantly improved performance in Kolmogorov-Arnold networks, though not in all models.

In the other parts of the study, Sect. 2 provides information on data acquisition, statistical properties of the data, data preprocessing, machine learning models, the deep learning model, and autoencoders. Section 3 presents the evaluation metrics of the experiments, statistical tests, experimental setup, and the results of the experiments and tests. In Sect. 4, the results are interpreted, and potential future studies are discussed. Additionally, the Appendix details the search space used in hyperparameter optimization and the selected hyperparameters.

## Materials and methods

### Image dataset

Individuals of the species *Pontastacus leptodactylus* Eschscholtz, 1823 were obtained from local fishermen during the 2017 and 2018 fishing seasons in Eğirdir Lake, Beyşehir Lake, and Hirfanlı Lake. In this study, a total of 112 crayfish were examined, including 62 females and 50 males^35^. The specimens were transported to the laboratory for measurements such as weight (W), carapace length (CL), carapace width (Cw), abdomen length (AL), abdomen width (Aw), cheliped length (ChlL), cheliped width (Chw), and cheliped height (ChL). Additionally, the sex of the specimens was determined, and after the organism was inverted, at least five images were taken from both the top and bottom and recorded according to standard measurement specifications. A total of 1,277 images were used in the research. The sex of the crayfish was determined by examining specific anatomical features such as reproductive organs (gonopores, size, and shape of the abdomen, claspers, coloration, and size)^36^.

In the tabular dataset, the class ratio among the total of 112 samples was calculated as 0.806:1. First, to ensure a balanced evaluation of the dataset, the data was shuffled according to the 10-fold cross-validation method, ensuring a balanced class distribution in each fold. Using this method, the distribution of female and male samples presented in Table 1 was obtained.

**Table 1:** Female - Male count distribution for each fold.

Sex	Fold 1	Fold 2	Fold 3	Fold 4	Fold 5	Fold 6	Fold 7	Fold 8	Fold 9	Fold 10
Female	7	7	6	6	6	6	6	4	7	7
Male	5	5	5	5	5	5	5	7	4	4

After the balancing process, missing values were handled using mean, median, mode, and the k-nearest neighbors algorithm with the five nearest neighbors as a hyperparameter. As a result, four different tabular datasets were created. In these datasets, outliers were corrected using the interquartile range (IQR) method for each numerical feature. In the IQR method, outliers are defined as values that fall outside the lower or upper boundary. These values are replaced with the closest boundary. Equations 1 and 2 are used to calculate the lower and upper boundaries, respectively.

1 $$\:Lower\:Boundary=\:{Q}_{1}-\:\left(IQR*Multiplier\:\right)$$

2 $$\:Upper\:Boundary=\:{Q}_{3}-\:\left(IQR*Multiplier\right)$$

$\:{Q}_{1}$ represents the value below which 25% of the data falls, while $\:{Q}_{3}$ represents the value below which 75% of the data falls. IQR is the difference between $\:{Q}_{3}$ and $\:{Q}_{1}$. In this study, the multiplier was set to 3. These operations were performed for each numerical feature.

In this study, the dataset created using Min-Max normalization was used as the fifth dataset. Since the dataset filled with mode demonstrated better performance in the cumulative total of accuracy metric results across all models compared to other datasets, Min-Max normalization was applied to it. The normalization process was performed using the MinMaxScaler class from the Scikit-learn library. This class applies the operations defined in Eqs. 3 and 4. As the feature range was set to [0,1], the data was scaled within this range.

3 $$\:{X}_{std}=\:\frac{X-\:{X}_{min}}{{X}_{max}-\:{X}_{min}}$$

4 $$\:{X}_{scaled}=\:{X}_{std}*\:\left(\mathrm{max}-\:min\right)+min$$

In this study, The MinMaxScaler class was used with its default hyperparameters. In this equation, $\:{X}_{min}$ and $\:{X}_{max}$ represent the minimum and maximum values of the corresponding feature, respectively. $\:{X}_{std}$ denotes the normalized values of the features. $\:{X}_{scaled}$ represents the transformed version of the normalized data based on the specified $\:min$and $\:max$ values. In this study, the data was normalized within the range of [0, 1].

Except for the dataset created with Min-Max normalization, data standardization was performed during the training and testing phases using the StandardScaler class from the Scikit-learn library. The data standardization process can be expressed by Eq. 5.

5 $$\:Z=\:\frac{x-u}{s}$$

In this study, the StandardScaler class was used with its default hyperparameters. In this equation, $\:u$ represents the mean of the training data, while $\:s$ denotes the standard deviation of the training data. For the standardization of the test data, the mean $\:u$ and standard deviation $\:s$ values obtained from the training data were used.

The image dataset of 112 specimens contains a total of 1,277 samples. Among these samples, 717 belong to female individuals, while 560 belong to male individuals. The class ratio in the dataset was calculated as 0.781:1. The dataset was split into 70% training and 30% testing, with this ratio being approximately maintained in both the training and test sets. The training set consists of a total of 895 samples, of which 501 are female and 394 are male. The test set contains a total of 382 samples, with 216 being female and 166 being male.

The image data was recorded in .jpg format with the RGB (red, green, and blue) color system, consisting of three channels and a resolution of 4608 × 3456 pixels. In this study, these images were converted to grayscale (one-channel) format and then resized to 28 × 28 pixels. The grayscale and resized images were transformed into tensor format and normalized with a mean of 0.5 and a standard deviation of 0.5. Using the preprocessed image data, training and test sets were obtained with the help of an autoencoder.

One of the main limitations of this study concerns potential variations in the image acquisition process. Although all samples were collected from three different lakes during the 2017–2018 fishing seasons, the dataset was created in collaboration with local fishermen. Therefore, it cannot be confirmed whether all images were captured using the same equipment or by the same operator. Such differences may have caused variations in lighting, shooting angle, or overall image quality, which could, in turn, affect the model’s ability to generalize to new conditions. Considering that real-world data are often collected by different people using different devices, it would be useful for future studies to examine how the proposed models perform under varying imaging setups and environmental conditions.

Differences in equipment and operators are commonly referred to in the literature as domain shift or device-induced variability, and are recognized as major factors that can hinder model generalization^37,38^. Previous research has shown that even when using the same network architecture, model performance can drop significantly if the data are collected with different cameras, scanners, or acquisition protocols^38^. For instance, Brown et al. (2024) reported that simply changing the camera used for image collection could alter classification outcomes^39^. Similarly, systematic reviews highlight that variations in acquisition conditions can lead to distributional shifts, ultimately impacting model performance^38^. From this perspective, the dataset used in our study may also have been affected by such variations in acquisition settings. To mitigate this limitation, future research could adopt season-based or location-based grouped validation strategies (e.g., leave-season-out or leave-location-out cross-validation), which help minimize data leakage and provide a more realistic assessment of model performance under real-world conditions^40^.

### General framework

**Fig. 1:**
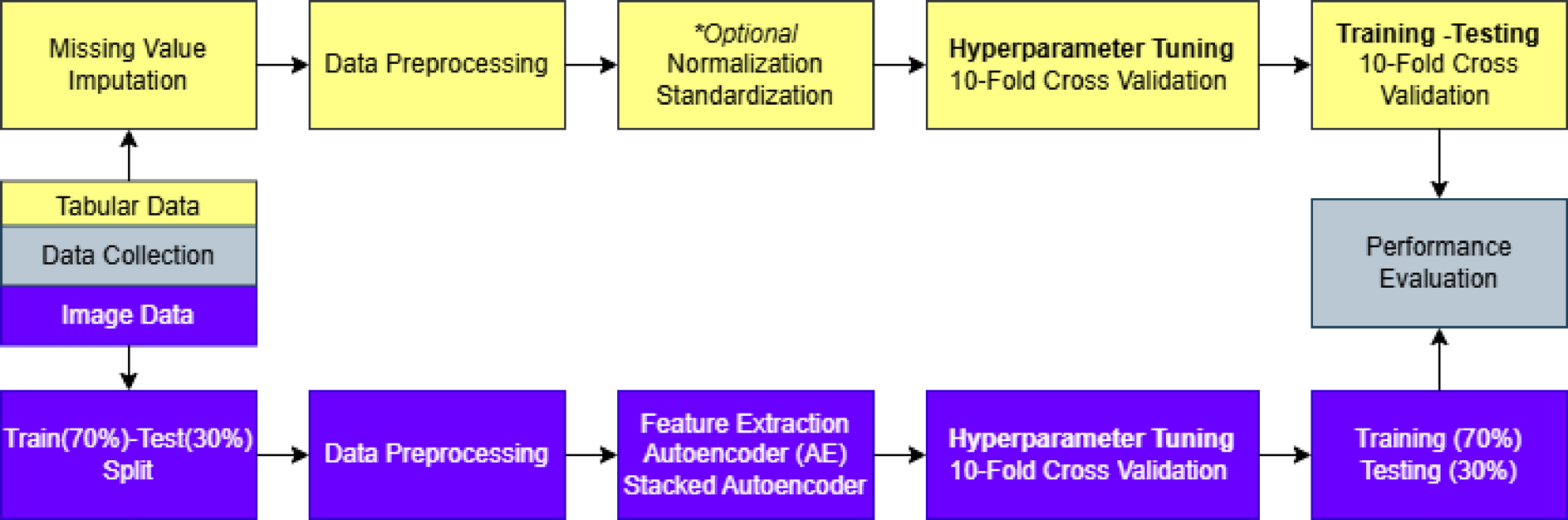
Overview of the proposed crayfish sex classification workflow.

**Fig. 2:**
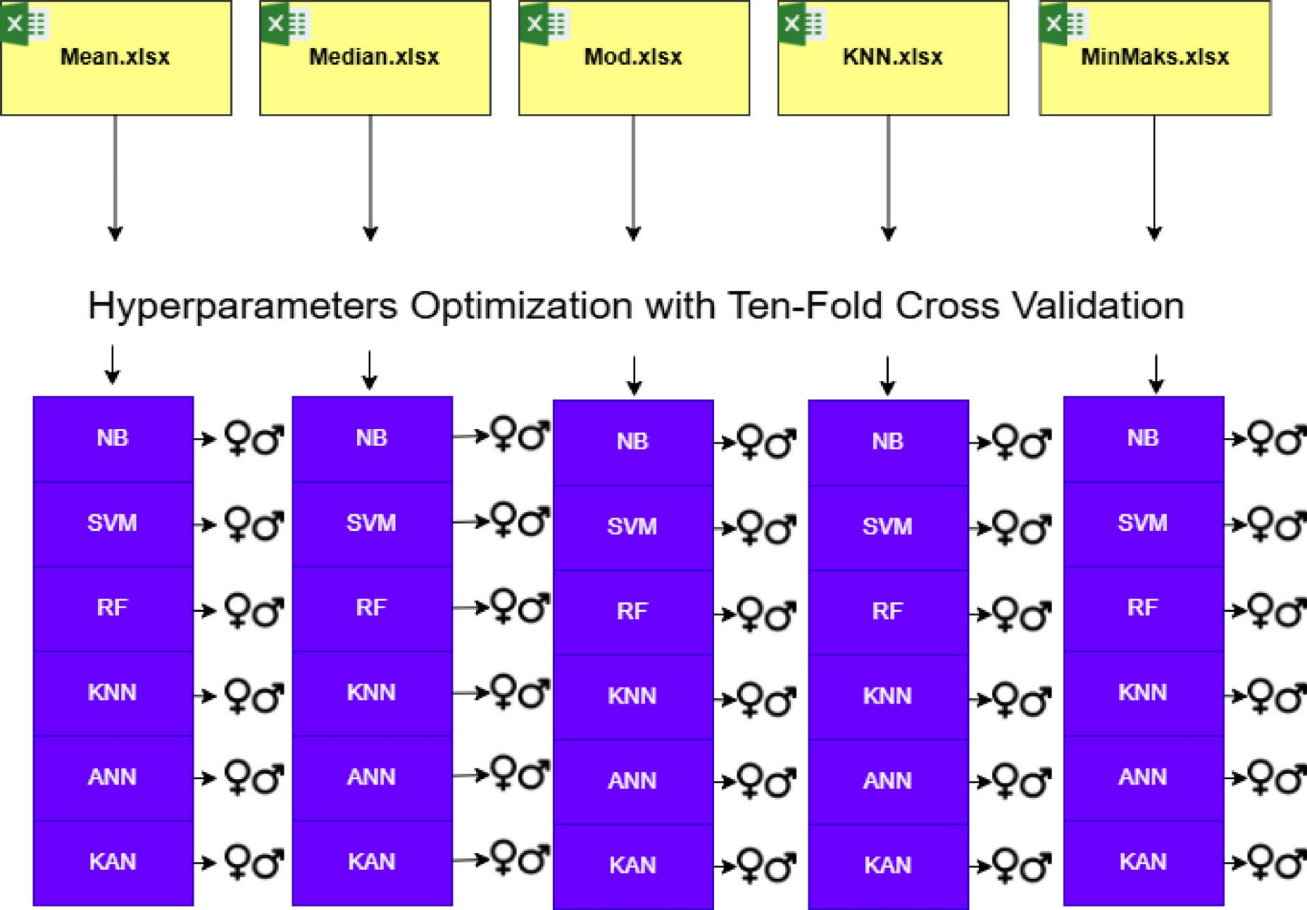
General framework for canonical machine learning and deep learning algorithms on tabular datasets.

**Fig. 3:**
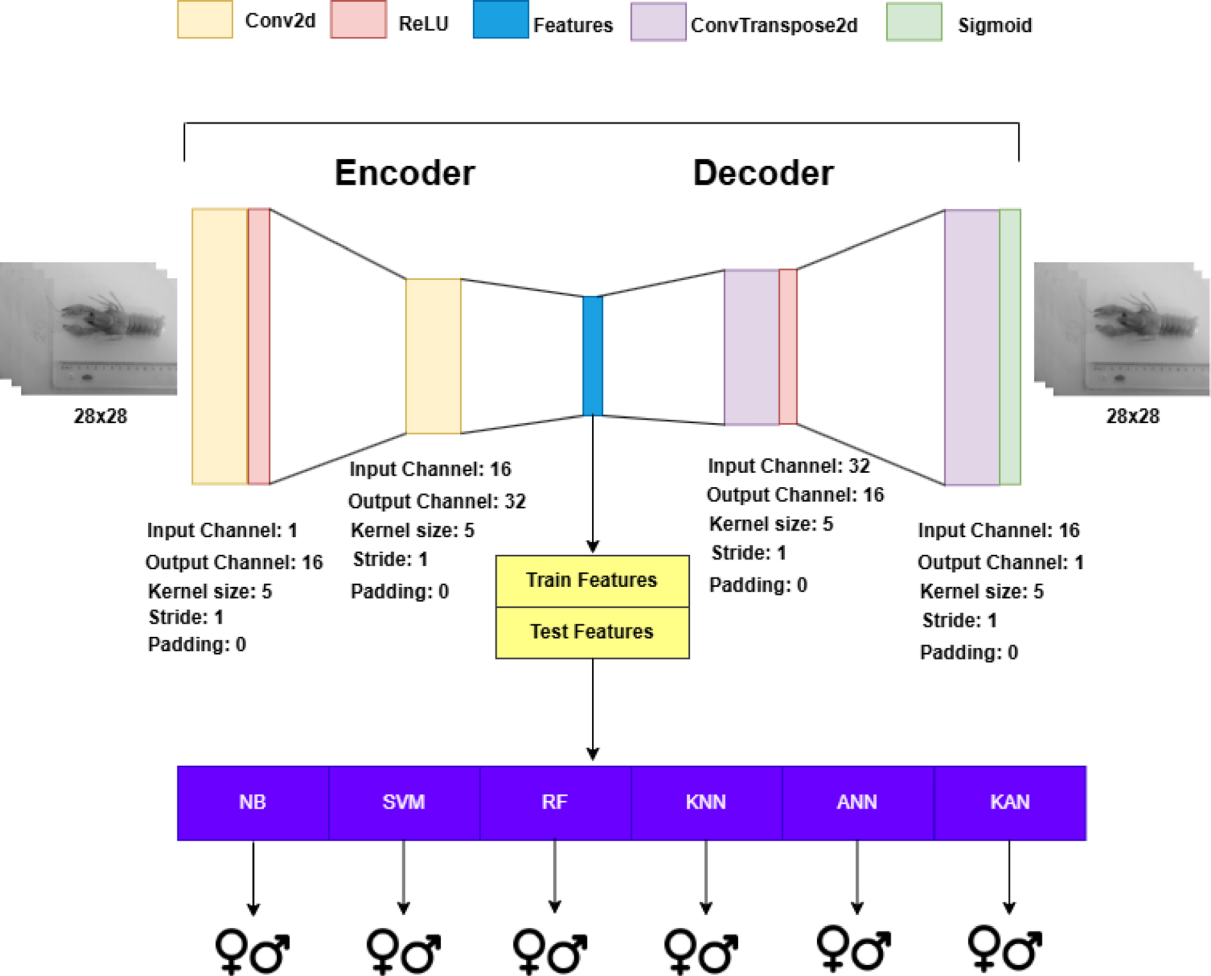
General framework for feature set obtained from the autoencoder.

**Fig. 4:**
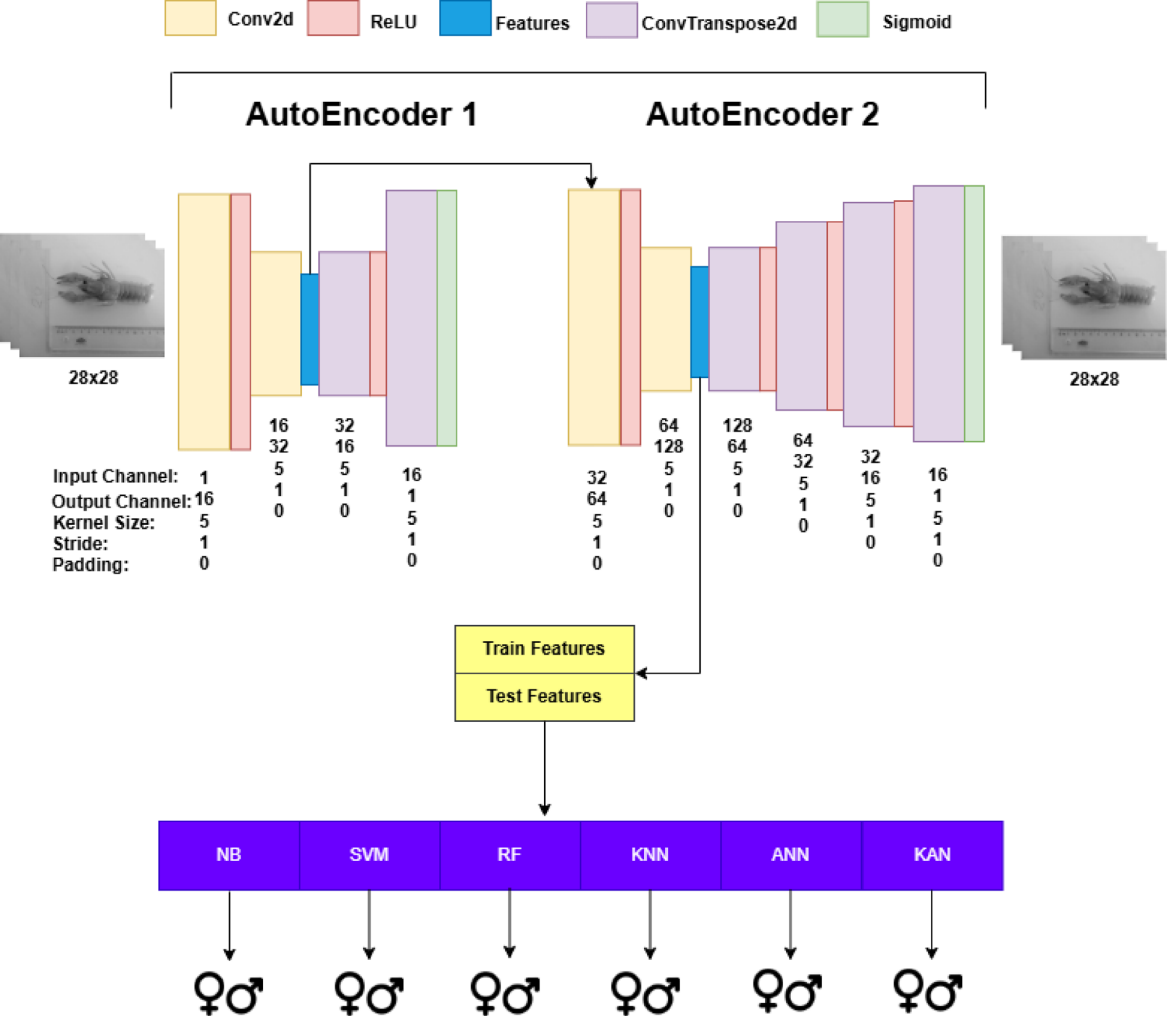
General framework for feature set obtained from the stacked autoencoders.

In this study, machine learning and deep learning algorithms were trained and tested on tabular datasets generated during the data preprocessing stage, as described in Sect. 2.1, to perform binary classification of crayfish as male or female. Additionally, using image data of crayfish, convolutional autoencoder and stacked convolutional autoencoder were employed to extract more abstract and meaningful features from the images. These newly extracted features were then used to train and test the same algorithms in a similar manner. Figure 1 illustrates the overall workflow of the proposed study, summarizing the main stages from data collection to model evaluation for both tabular and image datasets. The three general frameworks utilized in this study are presented in Figs. 2 and 3, and 4.

Figure 2 presents the overall workflow designed for the experiments conducted on the tabular datasets. The workflow consists of three main steps. In the first step, data preparation and preprocessing were performed, where the .*xlsx* files were generated using different imputation methods such as mean, median, mode, and K-Nearest Neighbors, as described in Sect. 2.1 Data. In the second step, hyperparameter optimization was carried out to determine the most appropriate hyperparameters for each machine learning algorithm using the Ten-Fold Cross-Validation method. In the third step, the models were trained and evaluated, and the details of this process are provided in Sect. 3.3 Experimental Setups. Once the optimal hyperparameter sets were identified, each fold was used as a test set to assess the overall model performance, as specified in Table 1. This process was repeated ten times, ensuring that all data were used for both training and testing phases.

Within the framework presented in Fig. 3, a two-layer encoder-decoder architecture was employed for a convolutional autoencoder. This autoencoder was trained using the preprocessed image training dataset described in Sect. 2.1. For each layer, the number of input and output channels, kernel size, stride, and padding parameters were specified. After training, the trained weights were utilized to generate feature sets through the autoencoder. Once the feature sets were obtained, hyperparameter optimization was conducted using the Ten-Fold Cross Validation method on the training feature dataset for each algorithm. After determining the optimal hyperparameters, the models were trained with these parameters and subsequently tested.

The architecture shown in Fig. 4 has been improved by adding an additional autoencoder to the previous design. The feature set obtained from the encoder layer of the first autoencoder is used as the input data for the second autoencoder. The number of encoder layers in the second autoencoder is the same as in the first one; however, the number of input and output channels differs. Additionally, the decoder structure of the second autoencoder has been redesigned differently from the first one to accommodate an input with 128 channels. In this context, the training and feature extraction procedure of the autoencoder remains the same as in Fig. 3.

### Canonical machine learning methods

Support Vector Machines (SVM) find the optimal hyperplane to separate data points in classification problems^41^. The distance between the hyperplane and the data samples called support vectors is the margin. The hyperplane with the widest margin between classes is achieved with the optimal margin. During model training, support vectors are identified, and the hyperplane is optimized accordingly^42^. For linearly non-separable datasets, higher-dimensional space is created using functions. The most commonly used functions for this purpose are polynomial and radial basis functions^43^.

During the classification process of an input vector, it is compared with support vectors and mapped to a high-dimensional space through a kernel function. The values obtained from the function are weighted using Lagrange multipliers to predict the class to which the input belongs^41^.

Naïve Bayes (NB) is a probabilistic model that utilizes Bayes’ theorem. It assumes that each feature in the dataset is independent. The posterior probability is the probability that a given example belongs to a specific class, given its feature vector. In calculating this probability, prior probability, conditional probability, and evidence values are used. The prior probability represents the probability of an example belonging to a class. The conditional probability is the probability of a feature vector occurring given the class information. Here, the assumption of conditional independence of features is applied, and the conditional probabilities of each feature are multiplied. Evidence, on the other hand, is the probability of a feature vector occurring without considering class information^44^.

The NB classifier selects the highest posterior probability as its prediction. This probability is calculated by dividing the product of the prior probability and the conditional probability by the evidence probability. Different methods can be used to compute conditional probability. For data with continuous values, the Gaussian kernel is used. In this case, the standard deviation and mean of the features are calculated from the training data and applied accordingly^44^.

The K-Nearest Neighbors (KNN) method uses the nearest neighbor rule on pre-labeled data to classify a given sample. The value of K represents the number of neighbors considered in the labeled dataset. The sample is assigned to the class of the majority of its nearest neighbors^45^. KNN-based density estimation offers an alternative approach to the fixed volume approach used in kernel-based methods. In the data space, while estimating local densities, the K value is kept constant, and the local density volume is estimated^46^.

In the KNN method, there is no active training process for parameter optimization. The constructed model only uses labeled training data. When assigning a class to a given sample, the distances of K data points in the training set are measured based on specific distance metrics. Euclidean, Manhattan, Minkowski, and Hamming distances are among these metrics^47^.

Multilayer Perceptrons (MLP) are deep learning models also referred to as deep feedforward networks^48^. These architectures include a function that processes the input and is used as a classification model. Typically, the input represents the feature vector of the instance to be classified. Instead of making a classification based on the output of a single function, the output of a function applied to the input vector can serve as the input to another function. Similarly, the output of this function can be used as the input to yet another function. This chain structure continues depending on the network design, and the number of such functions determines the depth of the network. Each function in this structure corresponds to a layer, and the final layer, which determines the network’s output, is called the output layer.

Each layer consists of processing units, known as neurons, that operate in parallel. These neurons are connected to the neurons in the previous layer through weighted connections. The information received by a neuron is obtained by multiplying the input vector from the previous layer with the connection weights and summing the resulting values. The obtained value is then processed by the neuron’s activation function to produce the final neuron output^48^.

The training of an MLP is conducted to align the outputs corresponding to input feature vectors with the true labels. This process is achieved by appropriately optimizing the connection weights within the network. First, the feedforward process is performed to obtain the predicted values at the output layer. Then, these predictions are compared with the true labels to calculate an error value. This error is expressed as a single cost value through a predefined loss function. Subsequently, the weights are updated using the backpropagation algorithm and gradient-based optimization methods based on this cost value. This process iterates until a predefined stopping criterion is met^48^.

Random Forest (RF) consists of multiple decision trees and utilizes the ensemble learning technique^49^. In RF, each decision tree has a vote for class prediction, and the final class prediction is determined based on this voting process. Each tree has a different structure, and the correlation between trees is reduced. Using a method called bagging, random samples from the training set are selected for each tree. During the splitting process in decision trees, the use of randomly selected features makes the model more robust to noise, enhancing its generalization ability.

After creating datasets for each tree using bagging, each decision tree is trained using Classification and Regression Tree (CART) algorithms^50^. Different types of metrics can be used to determine the feature that enables the split in tree construction. These metrics include Gini impurity, information gain, and mean squared error.

### Stacked convolutional autoencoders

An autoencoder is a type of neural network composed of an encoder and a decoder mechanism^48^. The encoder transforms the input of the network into a lower-dimensional representation. The decoder reconstructs this representation to reproduce it in a way similar to the network’s input. Autoencoders are architectures that do not directly copy the input data to the output. The learning process of an autoencoder involves minimizing the loss function between the input data and the reconstructed data.

Convolutional neural networks (CNNs) are used for classification by extracting features from image data through convolutional layers, subsampling layers, and classification layers^51^. Convolutional layers extract features such as edges and shapes from image data to create representations. The subsampling process further reduces the size of feature maps, lowering computational cost. The classification layer uses the extracted features to perform classification.

Convolutional Autoencoders (CAEs) preserve the 2D structure of images and learn local features, unlike fully connected autoencoders^52^. In traditional autoencoders, each feature is spread across the entire image, whereas in CAEs, weight sharing through kernel usage enables a parameter-efficient approach. This allows the model to discover repeating patterns, obtain better representations, and reconstruct images in small patches, making it more effective in computer vision models. Multiple CAEs can be stacked together to form Stacked CAE (SCAE) structures. These autoencoders can serve as feature extraction mechanisms, providing datasets for classifiers such as SVM.

Stacked Convolutional Autoencoders (SCAEs) have demonstrated superior performance in feature extraction and classification tasks compared to Stacked Denoising Autoencoders (SDAs). Specifically, experiments on the MNIST dataset and real-world video data have shown that SCAEs produce more effective feature representations^53^. The SDAs method aims to enhance the robustness of autoencoders by adding artificial noise to the input data. Based on this approach, a different study combined SDA and SCAE techniques in a hybrid manner to develop the Stacked Convolutional Denoising Autoencoder (SCDAE) model. SCDAE has improved feature representations on datasets such as MNIST and CIFAR-10, thereby enhancing the performance of classifier models^54^. The Stacked Convolutional Sparse Autoencoder (SCSAE) model, based on the idea that neurons are not active at a certain time, was developed and achieved successful results on the CIFAR-10 and MNIST datasets^55^. SCAEs can be used as an initialization mechanism to improve the feature extraction performance of CNN models. Instead of initializing CNNs with random weights, starting training with convolutional kernels learned by SCAE significantly enhances the classification performance of traditional CNNs^56^.

### Kolmogorov Arnold networks

The Kolmogorov-Arnold Network (KAN) is an artificial neural network designed based on the Kolmogorov-Arnold theorem, which decomposes complex functions into univariate components. Particularly effective in classification and regression tasks, KAN is distinguished by its capacity to learn linear and nonlinear components separately, making it well-suited for modeling high-dimensional data. Unlike traditional neural networks, KAN employs B-spline-based transformations to decompose input features into multiple subcomponents, enabling it to capture both global and local variations, thereby enhancing generalization performance.

The fundamental principle of KAN is rooted in Kolmogorov’s universal representation theorem, which asserts that any continuous function can be expressed as a composition of univariate functions. Leveraging this concept, KAN applies B-spline interpolation to decompose multivariate input features, facilitating more effective modeling. Two key hyperparameters underpin its architecture: grid size (G), which determines the partitioning of the data space, and spline degree (S), which defines the nonlinear transformation capacity of each segment. This structure offers a significant advantage by enabling flexible modeling of nonlinear relationships in high-dimensional data spaces^57^.

KAN’s network architecture differs from conventional feedforward networks (MLPs). The input layer consists of neurons corresponding to each feature in the dataset, while the hidden layers incorporate B-spline-based transformation mechanisms that integrate linear and nonlinear transformations. The output layer utilizes sigmoid or softmax activation functions for classification tasks. By replacing traditional activation functions with adaptive B-spline transformations, KAN enables a more flexible and interpretable learning process, effectively mitigating common deep learning challenges such as vanishing gradients and saturation.

Although machine learning techniques such as Support Vector Machines (SVMs) and Artificial Neural Networks (ANNs) effectively learn nonlinear decision boundaries, KAN’s spline-based structure provides a more detailed feature transformation, leading to improved accuracy. Ultimately, spline-based transformations enhance classification performance by distinguishing linear and nonlinear components separately, while also increasing the model’s sensitivity to data distribution^58^.

## Results

### Evaluation metrics

In this study, the performance of different sex classification models was evaluated using metrics such as accuracy, sensitivity, precision, specificity, F1 score, and Matthews correlation coefficient (MCC). These performance metrics are calculated based on the true positive (TP), true negative (TN), false positive (FP), and false negative (FN) values obtained from the classification results.

According to the prediction performance of a classification model, correctly predicting samples with the actual value of male as male is defined as TP, while incorrectly predicting samples with the actual value of female as male is referred to as FP. Similarly, correctly predicting samples with the actual value of female as female is defined as TN, whereas incorrectly predicting samples with the actual value of male as female is defined as FN.

Accuracy is the ratio of the correct predictions made by the model to the total number of samples. Recall is the model’s ability to correctly identify the samples that should be predicted as positive. Specificity is the model’s ability to correctly identify the samples that should be predicted as negative. Precision is the proportion of correctly predicted positive samples among all samples predicted as positive. The F1 score is a metric that balances the performance of the model in terms of precision and recall. The MCC metric is a measure that considers all values in the confusion matrix. The metric calculations are presented in Table 2.

**Table 2:** Equations for metric calculations.

Accuracy	$\:\frac{TP\:+\:TN}{TP\:+\:FP\:+\:TN\:+\:FN}$
Sensitivity	$\:\frac{TP}{TP\:+\:FN}$
Specificity	$\:\frac{TN}{TN\:+\:FP}$
Precision	$\:\frac{TP}{TP\:+\:FP}$
F1 score	$\:\frac{2\times\:\:Precision\:\times\:\:Sensitivity}{Precision\:+\:Sensitivity}$
MCC	$\:\:\frac{TP\:\times\:\:TN\:-\:FP\:\times\:\:FN}{\sqrt{(TP\:+\:FP)\times\:(TP\:+\:FN)\times\:(TN\:+\:FP)\times\:(TN+FN)}}$

### Statistical tests

#### Wilcoxon test

The Wilcoxon signed-rank test is a non-parametric statistical test method used to examine the significant difference in classification prediction accuracies between two models. The formula used in this method is given in Eq. 6.

6 $$\:W=min\left({\sum\:}_{i:{d}_{i}>0}{R}_{i},{\sum\:}_{i:{d}_{i}<0}{R}_{i}\right)$$

In the equation, $\:{d}_{i}$ represents the difference between predictions for the same sample, while $\:{R}_{i}$​ denotes the ranking of absolute differences. The W value is calculated by comparing it with the critical values from the Wilcoxon signed-rank distribution table. Additionally, the p-value can be computed to determine statistical significance.

#### McNemar’s test

The non-parametric McNemar statistical method uses model predictions to determine the statistical significance of the performance difference between two classification models. In a binary classification task, positive samples can be labeled as 1 and negative samples as 0. The models to be compared can be tested on $\:n$ samples, denoted as CM1 and CM2, respectively. McNemar’s test focuses on the samples where the models make different classification predictions. To perform the McNemar test, a contingency table is constructed, in which the values $\:{n}_{11}$​, $\:{n}_{10}$​, $\:{n}_{01}$​, and $\:{n}_{00}$ are calculated. $\:{n}_{11}$ represents the number of samples classified as positive by both models, while $\:{n}_{00}$ represents the number of samples classified as negative by both models. $\:{n}_{10}$ represents the number of samples classified as positive by CM1 but negative by CM2, whereas $\:{n}_{01}$​ represents the number of samples classified as negative by CM1 but positive by CM2.

The McNemar test statistic uses the $\:{n}_{10}$ and $\:{n}_{01}$ values from this table and is calculated as shown in Eq. 7.

7 $$\:{X}^{2}=\:\frac{{\left(\left|{n}_{10}-\:{n}_{01}\right|-1\right)}^{2}}{{n}_{10}+\:{n}_{01}}$$

Statistical significance can also be determined by calculating the p-value.

### Experimental setup

In the five tabular datasets described in the preparation phase in Sect. 2.1 Data, Ten-Fold Cross Validation was performed both during hyperparameter optimization and after determining the optimal hyperparameters in the training and testing phases. During hyperparameter optimization, models were trained and tested using each combination set specified in the hyperparameter set, with one fold used for testing while the remaining folds were used for training. The best hyperparameter set was determined using the accuracy metric. Afterward, the model training process was repeated ten times, where nine folds were used for training and one fold for testing. The folds used in each iteration were different. As a result, a confusion matrix with 112 samples was generated for each model. For NB models, hyperparameter optimization was not performed, and the models were trained and tested with default settings. In all datasets, except for the one created using Min-Max normalization, the data standardization process shown in Eq. 5 was applied during both the hyperparameter optimization and the training and testing phases.

To generate datasets through feature extraction using the autoencoder, the autoencoder models were first trained. The hyperparameters of the convolutional layers are provided in Figs. 3 and 4. While preparing the train and test sets, the DataLoader object was used with a batch size of 16 and shuffle set to True. The Adam optimizer was used with a learning rate of 0.001 and an epoch count of 100. However, an early stopping method was applied using patience set to 5 and delta set to 0.001. After completing the autoencoder training with the training data, the feature extraction method was executed using the learned weights, and the extracted features were used to generate training and test datasets for machine learning and deep learning models.

After obtaining the training and test data through feature extraction, the procedure is similar to the other four datasets, except for the dataset created using Min-Max normalization. The difference here is that the best hyperparameters are determined using Ten-Fold Cross Validation on 70% of the training data. Then, the model is trained on the training data using the selected hyperparameters. The trained model is tested on the remaining 30% of the test data, and a confusion matrix is generated. The sizes of the training and test sets extracted from the autoencoder architecture shown in the framework in Fig. 3 are (895, 12800) and (382, 12800), respectively. The sizes of the training and test sets extracted from the stacked autoencoder architecture shown in the framework in Fig. 4 are (895, 18432) and (382, 18432), respectively.

The experiments within the framework shown in Fig. 2 were conducted using Python-based Scikit-learn, NumPy, and Pandas tools^59–61^. The models were used in the experiments with the Scikit-learn library. These models were derived from the MLPClassifier, KNeighborsClassifier, GaussianNB, RandomForestClassifier, and SVC classes. The StandardScaler class was used for data standardization, and the MinMaxScaler class was used for Min-Max normalization. GridSearchCV and Pipeline classes were utilized for hyperparameter optimization. The Pipeline includes standardization and the model. NumPy and Pandas libraries were used for processing and data analysis. The autoencoder architecture shown in the frameworks in Figs. 3 and 4 was implemented using the PyTorch library^62^. The convolutional layers were derived from the nn.Conv2d and nn.ConvTranspose2d classes. The nn.ReLU and nn.Sigmoid classes were used for activation function layers. The custom classes written for the autoencoders inherited from the nn.Module class. The optim.Adam class was used for autoencoder training, the nn.MSELoss class for loss computation, and the DataLoader class for data handling.

The KAN model was developed using the specialized KANLinear class, which facilitates spline-based nonlinear transformations. The architecture comprises six layers, with an input layer of 11 neurons, followed by hidden layers containing 256, 128, 64, and 32 neurons, respectively, and a sigmoid activation function in the output layer. During the learning process, input features undergo adaptive B-spline transformations, which integrate both spline-based and linear components to effectively capture global and local relationships within the data.

To optimize the model, a Grid Search method was employed to identify optimal hyperparameters, selecting the most effective parameters based on accuracy metrics. Additionally, the Stochastic Gradient Descent (SGD) and Adam optimization algorithms were compared, with experimental results indicating that the Adam optimizer achieved superior accuracy. Consequently, the Adam algorithm was adopted for model training.

To mitigate overfitting, an early stopping mechanism was implemented, ensuring the training process was halted when further improvement was no longer observed. Furthermore, the ReduceLROnPlateau algorithm was applied to dynamically adjust the learning rate, enhancing the model’s adaptability and convergence efficiency.

The hyperparameter sets used in the frameworks in Figs. 2, 3, and 4, as well as the training hyperparameters obtained from the optimization process, are provided in the appendix of the paper. For MLP, KNN, GNB, RF, and SVM, the remaining hyperparameters are the default hyperparameters of Scikit-learn version 1.5.0. The hyperparameter optimization of KAN models was also performed using GridSearchCV, and the hyperparameter details are provided in the appendix of the paper.

### Experimental results

The experimental results obtained on the datasets shown in the framework provided in Fig. 2 are presented in Tables 3, 4, 5, 6 and 7. Based on the results obtained from all tabular datasets, the KAN model achieved the best performance across all metrics. In gender classification, accuracy is a relatively more important metric compared to others, and SVM was the second-best performing model in this regard. The methods used for handling outliers, including mean, median, mode, and KNN algorithms, have influenced model performances. The sum of the accuracy columns for each table is 4.597, 4.608, 4.625, and 4.58 for Tables 3, 4, 5 and 6, respectively. The results of experiments using Min-Max normalization instead of standardization are presented in Table 7, where the total accuracy is 4.626. However, the performance of the best models, KAN and SVM, has decreased compared to the results in Table 5. On the other hand, the accuracy performance of the distance-based KNN method has increased by approximately 4%.

**Table 3:** Experimental results with data filled using mean and standardized.

Model	Accuracy	Recall	Specificity	Precision	F1-score	MCC
MLP	0.804	0.800	0.807	0.769	0.784	0.605
KNN	0.705	0.660	0.742	0.674	0.667	0.403
NB	0.571	0.360	0.742	0.529	0.429	0.110
RF	0.696	0.580	0.790	0.691	0.630	0.380
SVM	0.821	0.780	0.855	0.813	0.796	0.638
KAN	1.0	1.0	1.0	1.0	1.0	1.0

**Table 4:** Experimental results with data filled using median and Standardized.

Model	Accuracy	Recall	Specificity	Precision	F1-score	MCC
MLP	0.795	0.720	0.855	0.800	0.758	0.583
KNN	0.741	0.700	0.774	0.714	0.707	0.475
NB	0.589	0.380	0.758	0.559	0.452	0.149
RF	0.688	0.580	0.774	0.674	0.624	0.362
SVM	0.813	0.760	0.855	0.809	0.784	0.619
KAN	0.982	0.980	0.982	0.980	0.980	0.964

**Table 5:** Experimental results with data filled using mode and Standardized.

Model	Accuracy	Recall	Specificity	Precision	F1-score	MCC
MLP	0.786	0.720	0.839	0.783	0.750	0.565
KNN	0.714	0.640	0.774	0.696	0.667	0.419
NB	0.616	0.400	0.790	0.606	0.482	0.208
RF	0.688	0.580	0.774	0.674	0.624	0.362
SVM	0.830	0.820	0.839	0.804	0.812	0.658
KAN	0.991	1.0	0.984	0.980	0.990	0.982

**Table 6:** Experimental results with data filled using KNN and standardized.

Model	Accuracy	Recall	Specificity	Precision	F1-score	MCC
MLP	0.804	0.800	0.807	0.769	0.784	0.605
KNN	0.732	0.660	0.790	0.717	0.688	0.455
NB	0.571	0.300	0.790	0.536	0.385	0.104
RF	0.661	0.560	0.742	0.636	0.596	0.307
SVM	0.830	0.820	0.839	0.804	0.812	0.658
KAN	0.982	1.0	0.968	0.962	0.980	0.965

**Table 7:** Experimental results with data filled using mode – Min-Max normalized.

Model	Accuracy	Recall	Specificity	Precision	F1-score	MCC
MLP	0.813	0.800	0.823	0.784	0.792	0.622
KNN	0.750	0.660	0.823	0.750	0.702	0.491
NB	0.616	0.400	0.790	0.606	0.482	0.208
RF	0.661	0.540	0.758	0.643	0.587	0.306
SVM	0.813	0.800	0.823	0.784	0.792	0.622
KAN	0.973	0.960	0.984	0.98	0.970	0.946

The results obtained from the autoencoder framework in Fig. 3 and the stacked autoencoder framework in Fig. 4 are presented in Tables 8 and 9, respectively. On the feature datasets extracted using the autoencoder, SVM achieved the best performance in terms of accuracy, while the MLP model showed the second-highest performance. The performance of the KAN model, however, decreased in experiments conducted on feature sets extracted from image data. On the higher-dimensional feature dataset extracted using stacked autoencoders, the KAN model improved its accuracy performance by 3%, achieving the best results. Although the SVM model experienced a 4% decrease in accuracy performance, it still demonstrated the second-best performance.

**Table 8:** Experimental results on feature sets extracted using Autoencoder.

Model	Accuracy	Recall	Specificity	Precision	F1-score	MCC
MLP	0.819	0.813	0.824	0.780	0.797	0.635
KNN	0.801	0.759	0.833	0.778	0.768	0.594
NB	0.631	0.705	0.574	0.560	0.624	0.278
RF	0.778	0.705	0.833	0.765	0.734	0.544
SVM	0.840	0.855	0.829	0.793	0.823	0.680
KAN	0.780	0.795	0.769	0.725	0.759	0.560

**Table 9:** Experimental results on feature sets extracted using stacked autoencoders.

Model	Accuracy	Recall	Specificity	Precision	F1-score	MCC
MLP	0.783	0.771	0.792	0.740	0.755	0.560
KNN	0.796	0.759	0.824	0.768	0.764	0.584
NB	0.636	0.681	0.602	0.568	0.619	0.280
RF	0.780	0.711	0.833	0.766	0.738	0.550
SVM	0.806	0.807	0.806	0.761	0.784	0.609
KAN	0.819	0.807	0.829	0.784	0.795	0.634

Wilcoxon test and McNemar’s test were performed on the model results obtained from the Min-Max normalized tabular dataset and the datasets extracted from autoencoders. The Min-Max normalized dataset was selected because it had the highest total accuracy in the first framework shown in Fig. 2. The results of the Wilcoxon test and McNemar’s test are presented in Tables 10, 11, 12, 13, 14 and 15, respectively. A 5% threshold was chosen, and if there was a statistically significant difference between the two models, the corresponding value was highlighted in bold. Additionally, an arrow was added to the relevant cell to indicate the model that performed better in terms of accuracy.

Statistical analysis of the Wilcoxon test results indicates that, based on the tabular dataset results in Table 10, there is a significant difference between KAN and GNB, MLP and GNB, and SVM and GNB. Accordingly, these three models performed better than the GNB model. Based on the test results of the dataset obtained from the autoencoder in Table 11, there is a significant difference between GNB and all other models, as well as between KAN and RF, KAN and KNN, MLP and RF, SVM and KNN, and SVM and RF. The results show that the GNB model performed worse than the other models. The RF model also showed lower performance compared to the KAN, SVM, and MLP models. While the KNN model performed worse than the SVM model, it performed better than the KAN model. Based on the test results of the dataset obtained from the stacked autoencoder in Table 12, the GNB model showed the lowest performance compared to all other models. The RF model also performed worse than the KAN, MLP, and SVM models.

Statistical analysis of the McNemar’s test results revealed similar outcomes to those of the Wilcoxon tests across all experiments. The same model comparisons can be reiterated for this test as well. The most significant difference in these tests is that, in the dataset obtained using the autoencoder, the p-value calculated from the predictions of the SVM and KNN models is slightly above 0.05. Other statistical significances (or differences) observed in the Wilcoxon test have been confirmed in the same manner.

**Table 10:** Wilcoxon test results for the dataset filled using mode - Min-Max normalized.

*p* < 0.05						
Classifier	KAN	MLP	SVM	KNN	RF	NB
KAN		0.655	0.655	0.336	0.250	**0.014 ←**
MLP			1.000	0.127	0.083	**0.002 ←**
SVM				0.144	0.083	**0.002 ←**
KNN					0.593	0.063
RF						0.083
NB						

**Table 11:** Wilcoxon test results for the feature set obtained from the autoencoder.

*p* < 0.05						
Classifier	KAN	MLP	SVM	KNN	RF	NB
KAN		0.241	0.686	**0.035 **↑	**0.001 ←**	**0.023 ←**
MLP			0.303	0.179	**0.006 ←**	**0.001 ←**
SVM				**0.044 ←**	**0.001 ←**	**0.009 ←**
KNN					0.299	**0.000 ←**
RF						**0.000 ←**
NB						

**Table 12:** Wilcoxon test results for the feature set obtained from the stacked autoencoder.

*p* < 0.05						
Classifier	KAN	MLP	SVM	KNN	RF	NB
KAN		0.732	0.336	0.399	**0.022 ←**	**0.001 ←**
MLP			0.631	0.305	**0.015 ←**	**0.019 ←**
SVM				0.157	**0.005 ←**	**0.033 ←**
KNN					0.275	**0.002 ←**
RF						**0.000 ←**
NB						

**Table 13:** McNemar’s test results for the dataset filled using mode and Min-Max normalized.

*p* < 0.05						
Classifier	KAN	MLP	SVM	KNN	RF	NB
KAN		0.824	0.824	0.442	0.324	**0.020 ←**
MLP			1.000	0.189	0.122	**0.003 ←**
SVM				0.210	0.122	**0.003 ←**
KNN					0.791	0.090
RF						0.122
NB						

**Table 14:** McNemar’s test results for the feature set obtained from the autoencoder.

*p* < 0.05						
Classifier	KAN	MLP	SVM	KNN	RF	NB
KAN		0.298	0.788	0.045 ↑	**0.001 ←**	**0.028 ←**
MLP			0.392	0.222	**0.009 ←**	**0.002 ←**
SVM				0.057	**0.001 ←**	**0.011 ←**
KNN					0.356	**0.000 ←**
RF						**0.000 ←**
NB						

**Table 15:** McNemar’s test results for the feature set obtained from the stacked autoencoder.

*p* < 0.05						
Classifier	KAN	MLP	SVM	KNN	RF	NB
KAN		0.864	0.442	0.470	**0.030 ←**	**0.013 ←**
MLP			0.749	0.362	**0.020 ←**	**0.023 ←**
SVM				0.195	**0.006 ←**	**0.042 ←**
KNN					0.326	**0.003 ←**
RF						**0.000 ←**
NB						

## Discussion

In this study, machine learning and deep learning models were trained and tested using tabular datasets created by handling missing values in different ways for the crayfish sex classification problem. Since sex classification was performed, although the results of different types of metrics are shared, accuracy can be used as the main evaluation criterion. For all models except the GNB model, key hyperparameters were selected and optimized. In the training and testing conducted with the selected hyperparameters for each model, the KAN model achieved the highest performance in terms of both the accuracy metric and other metrics compared to the other models. The exceptional performance of the KAN model can be attributed to its hybrid architectural framework, which seamlessly integrates both linear and nonlinear representations through B-spline-based transformations. By leveraging this adaptive transformation mechanism, KAN effectively captures global structural patterns while simultaneously preserving local variations within the data. This capability enhances the model’s expressiveness and generalization capacity, leading to more precise decision boundaries and improved classification performance across diverse feature distributions. Among the models, except for the KAN model, the SVM model achieved the highest accuracy performance. In the SVM model, the radial basis function (RBF) was used to linearly separate the data in a high-dimensional space. In the classification performed on the tabular datasets with 11 features in this study, the use of the RBF function was one of the key factors contributing to the better performance of the SVM model. The GNB model demonstrated lower performance in the applied metrics across all datasets compared to the other models. This suggests that the features in the datasets used in this study may have strong correlations. The dataset in which missing values were filled using the mode value of each feature performed better in terms of total accuracy. Therefore, a new dataset was created using Min-Max normalization, and the experiments were repeated. Additionally, data standardization was not applied to this dataset. In this dataset, an improvement of approximately 3% in the MLP model and 5% in the KNN model was observed in terms of accuracy. Since the KNN model is a method based on the distance between samples in the dataset, applying Min-Max normalization is a significant factor in the performance improvement. The dataset with Min-Max normalization has enabled better learning of weights in the MLP model and improved the effectiveness of activation functions.

In the second phase of the study, classification was performed by extracting features from image data. Two different architectures were designed for feature extraction. The first is the most basic autoencoder architecture, while the second is the stacked autoencoder architecture, created by combining two autoencoders side by side. Based on the datasets obtained from the autoencoder architecture, the SVM model achieved the highest accuracy performance at 84%. The second-best performance was achieved by the MLP model, while the GNB model showed the lowest performance. Except for the KAN model, all other models performed better on accuracy compared to their performance on tabular datasets. The higher-level features extracted by the autoencoders effectively improved these models’ performance. One of the most significant findings here is the substantial decline in the KAN model’s performance compared to its results on tabular datasets. The accuracy of the KAN model decreased by approximately 20%. Since the input layer of the KAN model is structured to match the number of features in the dataset, the amount of information entering the model’s input layer increases directly with high-dimensional datasets. However, the gradual reduction in the number of neurons in subsequent layers led to the loss of some features, resulting in information loss during the learning process. Specifically, the inability of KAN’s adaptive grid structure to effectively optimize in very high-dimensional feature spaces limited the model’s generalization ability, causing a drop in accuracy. Finally, the study aimed to enhance model performance by obtaining higher-level features using stacked autoencoders. However, the MLP and SVM models experienced a 3% decrease in accuracy performance. The high-dimensional dataset negatively impacted these models’ performance. Meanwhile, the KAN model achieved the best performance with an accuracy of approximately 82%, improving by 4%. This success is attributed to KAN’s spline-based transformations, which help reduce the influence of irrelevant components by learning only the most discriminative variables in high-dimensional feature spaces. The adaptive grid structure enabled the model to balance both global and local patterns in data distribution, minimizing the risk of overfitting. Consequently, KAN demonstrated stable and reliable predictions even on high-dimensional feature sets. In this context, KAN achieving an 82% accuracy rate can be attributed to its ability to minimize information loss while simultaneously creating flexible and generalizable decision boundaries.

In this study, Wilcoxon tests and McNemar’s tests were conducted to statistically evaluate the comparison of model predictions. Both tests yielded largely similar results. The KAN, MLP, and SVM models demonstrated statistically significantly better performance than the GNB model in all tests. In four tests related to the image dataset, the KAN, MLP, and SVM models showed statistically significantly better performance than the RF model.

## Conclusion

In this study, an original dataset containing both tabular and image data was used to address the crayfish sex classification problem, and classical machine learning as well as deep learning algorithms were compared. The effects of different missing-value imputation techniques, normalization procedures, and autoencoder-based feature extraction approaches on model performance were examined.

The obtained results indicated that the KAN model achieved the highest overall accuracy across both data types. The features extracted through the autoencoder architecture enhanced the performance of classical models, with the SVM and MLP models showing strong results on image-based datasets. With the stacked autoencoder architecture, the KAN model not only improved its performance but also outperformed the other models, demonstrating better adaptability in high-dimensional feature spaces. The applied statistical tests (Wilcoxon and McNemar) confirmed that the KAN, MLP, and SVM models achieved statistically significantly better performance compared to the GNB model.

Future research can improve the results by building upon this study. First, the amount of data can be increased through natural data collection methods or by generating synthetic data using deep learning-based generative approaches. Then, all the processes used in this study can be repeated in the same manner. Additional classification methods can be incorporated into the established frameworks, or the feature extraction mechanism used with autoencoders can be improved. Furthermore, the existing classification architectures in this study can be applied to other species where sex classification is important.

## Supplementary Information

Below is the link to the electronic supplementary material.

Supplementary Material 1

## Data Availability

The datasets generated and/or analysed during the current study are available in the Zenodo repository: https://doi.org/10.5281/zenodo.17516963. The source codes developed for the experiments are stored in a GitHub repository at https://github.com/yasinatilkan60/Crayfish-Sex-Identification.
