# Supplementary material for: Enhancing crayfish sex identification with Kolmogorov-Arnold networks and stacked autoencoders
Source: Sci Rep. 2025 Dec 30;16:3971. doi: 10.1038/s41598-025-34095-z (PMC12856012; doi:10.1038/s41598-025-34095-z)
Supplement: Supplementary file 1 — Supplementary Material 1 [file 41598_2025_34095_MOESM1_ESM.docx]

**Appendix: Hyperparameter Optimization Details**

**A. Hyperparameter Search Space for Framework 1**

| **Model** | **Hyperparameter** | **Range** |
| --- | --- | --- |
| MLP | hidden_layer_sizes | (32,), (64,), (128,), (32, 32), (64, 64), (128, 128), (32, 32, 32), (64, 64, 64), (128, 128, 128) |
| MLP | activation | relu, tanh, sigmoid |
| MLP | learning_rate_init | 0.001, 0.01, 0.1 |
| MLP | solver | adam, sgd, rmsprop |
| KNN | n_neighbors | list(range(1, 51, 2) |
| RF | max_depth | range(1,11) |
| SVM | svc_C | np.array([1.e-02, 1.e-01, 1.e+00, 1.e+01, 1.e+02, 1.e+03, 1.e+04, 1.e+05, 1.e+06, 1.e+07, 1.e+08, 1.e+09, 1.e+10]) |
| SVM | svc_gamma | np.array([1.e-09, 1.e-08, 1.e-07, 1.e-06, 1.e-05, 1.e-04, 1.e-03, 1.e-02, 1.e-01, 1.e+00, 1.e+01, 1.e+02, 1.e+03]) |
| KAN | hidden_layer_size | [11,256,128,64,32,1], [11, 128, 64, 32, 1],  [11, 32, 1],  [11, 128, 64, 32, 16, 1] |
| KAN | grid_size | (8, 7, 6, 5, 4] |
| KAN | spline_order | (5,4,3) |
| KAN | scale_base | (1.0, 2.0, 3.0) |
| KAN | scale_spline | (1.0, 2.0) |
| KAN | batch_size | (128, 64, 32) |
| KAN | optimizer | ("Adam", "SGD") |

**B. Selected Hyperparameters for Framework 1**

| **Dataset** | **Model** | **Hyperparamter** | **Value** |
| --- | --- | --- | --- |
| Mean.xlsx | MLP | hidden_layer_sizes | (64, 64) |
| Mean.xlsx | MLP | activation | relu |
| Mean.xlsx | MLP | learning_rate_init | 0.01 |
| Mean.xlsx | MLP | solver | sgd |
| Mean.xlsx | KNN | n_neighbors | 7 |
| Mean.xlsx | RF | max_depth | 10 |
| Mean.xlsx | SVM | C | 1000000000.0 |
| Mean.xlsx | SVM | gamma | 1e-06 |
| Mean.xlsx | KAN | hidden_layer_size | 11, 128, 64, 32, 1 |
| Mean.xlsx | KAN | grid_size | 7 |
| Mean.xlsx | KAN | learning_rate | 0.0005 |
| Mean.xlsx | KAN | spline_order | 5 |
| Mean.xlsx | KAN | scale_base | 1.0 |
| Mean.xlsx | KAN | scale_spline | 1.0 |
| Mean.xlsx | KAN | batch_size | 64 |
| Mean.xlsx | KAN | optimizer | Adam |
| Median.xlsx | MLP | hidden_layer_sizes | (32,) |
| Median.xlsx | MLP | activation | Relu |
| Median.xlsx | MLP | learning_rate_init | 0.001 |
| Median.xlsx | MLP | solver | adam |
| Median.xlsx | KNN | n_neighbors | 7 |
| Median.xlsx | RF | max_depth | 9 |
| Median.xlsx | SVM | C | 10.0 |
| Median.xlsx | SVM | gamma | 0.01 |
| Median.xlsx | KAN | hidden_layer_size | 11, 128, 64, 32, 1 |
| Median.xlsx | KAN | grid_size | 7 |
| Median.xlsx | KAN | learning_rate | 0.0001 |
| Median.xlsx | KAN | spline_order | 5 |
| Median.xlsx | KAN | scale_base | 1.0 |
| Median.xlsx | KAN | scale_spline | 1.0 |
| Median.xlsx | KAN | batch_size | 32 |
| Median.xlsx | KAN | optimizer | Adam |
| Mod.xlsx | MLP | hidden_layer_sizes | (128, 128) |
| Mod.xlsx | MLP | activation | Tanh |
| Mod.xlsx | MLP | learning_rate_init | 0.01 |
| Mod.xlsx | MLP | solver | adam |
| Mod.xlsx | KNN | n_neighbors | 7 |
| Mod.xlsx | RF | max_depth | 6 |
| Mod.xlsx | SVM | C | 1000.0 |
| Mod.xlsx | SVM | gamma | 0.001 |
| Mod.xlsx | KAN | hidden_layer_size | (11, 128, 64, 32, 1) |
| Mod.xlsx | KAN | grid_size | 7 |
| Mod.xlsx | KAN | learning_rate | 0.005 |
| Mod.xlsx | KAN | spline_order | 5 |
| Mod.xlsx | KAN | scale_base | 1.0 |
| Mod.xlsx | KAN | scale_spline | 1.0 |
| Mod.xlsx | KAN | batch_size | 64 |
| Mod.xlsx | KAN | optimizer | Adam |
| KNN.xlsx | MLP | hidden_layer_sizes | (64, 64, 64) |
| KNN.xlsx | MLP | activation | Relu |
| KNN.xlsx | MLP | learning_rate_init | 0.01 |
| KNN.xlsx | MLP | solver | sgd |
| KNN.xlsx | KNN | n_neighbors | 3 |
| KNN.xlsx | RF | max_depth | 9 |
| KNN.xlsx | SVM | C | 1000.0 |
| KNN.xlsx | SVM | gamma | 0.001 |
| KNN.xlsx | KAN | hidden_layer_size | (11, 128, 64, 32, 1) |
| KNN.xlsx | KAN | grid_size | 7 |
| KNN.xlsx | KAN | learning_rate | 0.0005 |
| KNN.xlsx | KAN | spline_order | 5 |
| KNN.xlsx | KAN | scale_base | 1.0 |
| KNN.xlsx | KAN | scale_spline | 1.0 |
| KNN.xlsx | KAN | batch_size | 64 |
| KNN.xlsx | KAN | optimizer | Adam |
| MinMaks.xlsx | MLP | hidden_layer_sizes | (64, 64) |
| MinMaks.xlsx | MLP | activation | Relu |
| MinMaks.xlsx | MLP | learning_rate_init | 0.001 |
| MinMaks.xlsx | MLP | solver | adam |
| MinMaks.xlsx | KNN | n_neighbors | 5 |
| MinMaks.xlsx | RF | max_depth | 6 |
| MinMaks.xlsx | SVM | C | 10000.0 |
| MinMaks.xlsx | SVM | gamma | 0.01 |
| MinMaks.xlsx | KAN | hidden_layer_size | 11, 128, 64, 32, 1 |
| MinMaks.xlsx | KAN | grid_size | 8 |
| MinMaks.xlsx | KAN | learning_rate | 0.005 |
| MinMaks.xlsx | KAN | spline_order | 4 |
| MinMaks.xlsx | KAN | scale_base | 1.0 |
| MinMaks.xlsx | KAN | scale_spline | 1.0 |
| MinMaks.xlsx | KAN | batch_size | 64 |
| MinMaks.xlsx | KAN | optimizer | Adam |

**C. Hyperparameter Search Space for Framework 2 and Framework 3**

| **Model** | **Hyperparameter** | **Range** |
| --- | --- | --- |
| MLP | hidden_layer_sizes | (32,), (64,), (128,), (32, 32), (64, 64), (128, 128), (32, 32, 32), (64, 64, 64), (128, 128, 128) |
| MLP | activation | relu, tanh |
| MLP | learning_rate_init | 0.001, 0.01 |
| MLP | solver | adam, sgd |
| KNN | n_neighbors | list(range(1, 51, 2)) |
| RF | max_depth | range(1, 11) |
| SVM | C | np.array([1.e-02, 1.e-01, 1.e+00, 1.e+01, 1.e+02, 1.e+03, 1.e+04, 1.e+05, 1.e+06, 1.e+07, 1.e+08, 1.e+09, 1.e+10]) |
| SVM | gamma | np.array([1.e-09, 1.e-08, 1.e-07, 1.e-06, 1.e-05, 1.e-04, 1.e-03, 1.e-02, 1.e-01, 1.e+00, 1.e+01, 1.e+02, 1.e+03]) |
| SAE-KAN (Framework 3) | hidden_layer_size | (18432, 1024, 512, 256, 128, 64, 32, 1)  (18432,512, 256, 128, 64, 32, 1)  (18432, 256, 128, 64, 32, 1)  (18432, 64, 32, 1) |
| SAE-KAN (Framework 3) | grid_size | (8, 7, 6, 5, 4) |
| SAE-KAN (Framework 3) | learning_rate | (0.0005, 0.005, 0.001, 0.0001) |
| SAE-KAN (Framework 3) | spline_order | (5,4,3) |
| SAE-KAN (Framework 3) | scale_base | (1.0, 2.0, 3.0) |
| SAE-KAN (Framework 3) | scale_spline | (1.0, 2.0) |
| SAE-KAN (Framework 3) | batch_size | (128,64,32) |
| SAE-KAN (Framework 3) | optimizer | Adam, SGD |
| AE-KAN (Framework 2) | hidden_layer_size | (12400, 1024, 512, 256, 128, 64, 32, 1)  (12400,512, 256, 128, 64, 32, 1)  (12400, 256, 128, 64, 32, 1)  (12400, 64, 32, 1) |
| AE-KAN (Framework 2) | grid_size | (8, 7, 6, 5, 4) |
| AE-KAN (Framework 2) | learning_rate | (0.0005, 0.005, 0.001, 0.0001) |
| AE-KAN (Framework 2) | spline_order | (5,4,3) |
| AE-KAN (Framework 2) | scale_base | (1.0, 2.0, 3.0) |
| AE-KAN (Framework 2) | scale_spline | (1.0, 2.0) |
| AE-KAN (Framework 2) | batch_size | (128, 64, 32) |
| AE-KAN (Framework 2) | optimizer | Adam, SGD |

**D. Selected Hyperparameters for Framework 2 and Framework 3**

| **Framework** | **Model** | **Hyperparamater** | **Value** |
| --- | --- | --- | --- |
| Framework 2 | MLP | hidden_layer_sizes | (128, 128) |
| Framework 2 | MLP | activation | relu |
| Framework 2 | MLP | learning_rate_init | 0.001 |
| Framework 2 | MLP | solver | sgd |
| Framework 2 | KNN | n_neighbors | 1 |
| Framework 2 | RF | max_depth | 9 |
| Framework 2 | SVM | C | 10.0 |
| Framework 2 | SVM | gamma | 0.0001 |
| Framework 2 | KAN | hidden_layer_size | 12400, 128, 64, 32, 1 |
| Framework 2 | KAN | grid_size | 7 |
| Framework 2 | KAN | learning_rate | 0.0005 |
| Framework 2 | KAN | spline_order | 5 |
| Framework 2 | KAN | scale_base | 1.0 |
| Framework 2 | KAN | scale_spline | 1.0 |
| Framework 2 | KAN | batch_size | 64 |
| Framework 2 | KAN | optimizer | Adam |
| Framework 3 | MLP | hidden_layer_sizes | (128, 128, 128) |
| Framework 3 | MLP | activation | relu |
| Framework 3 | MLP | learning_rate_init | 0.001 |
| Framework 3 | MLP | solver | sgd |
| Framework 3 | KNN | n_neighbors | 1 |
| Framework 3 | RF | max_depth | 10 |
| Framework 3 | SVM | C | 10.0 |
| Framework 3 | SVM | gamma | 0.0001 |
| Framework 3 | KAN | hidden_layer_size | 18432, 128, 64, 32, 1 |
| Framework 3 | KAN | grid_size | 7 |
| Framework 3 | KAN | learning_rate | 0.0005 |
| Framework 3 | KAN | spline_order | 5 |
| Framework 3 | KAN | scale_base | 1.0 |
| Framework 3 | KAN | scale_spline | 1.0 |
| Framework 3 | KAN | batch_size | 64 |
| Framework 3 | KAN | optimizer | Adam |
